# Preventing Surgical Site Infections: A Narrative Review of Antiseptic Solutions in the Operating Theatre

**DOI:** 10.7759/cureus.98016

**Published:** 2025-11-28

**Authors:** Ruqaiya Al-Habsi

**Affiliations:** 1 Surgery, The Royal London Hospital, London, GBR

**Keywords:** antiseptic solution, betadine, chlorhexidine gluconate, surgical site infection (ssi), ­wound healing

## Abstract

Surgical site infections (SSIs) are commonly encountered postoperatively and can lead to devastating effects to the patient in terms of prolonged recovery, extended hospital stay, additional morbidity and possibly mortality. For hospitals, it can increase cost burden, add to the bed crisis and prolong surgical waiting lists. Surgeons and scrub nurses use a variety of antiseptic agents to prepare their hands and patients' skin on the surgical site pre-operatively to help minimise the risk of infections. The objective of this narrative review was to retrieve and create a summary of existing evidence in the literature about the antiseptics used in surgery to prevent surgical site infections and highlight any possible limitations noted in the literature.

## Introduction and background

Surgical site infections (SSIs) are among the most commonly acquired hospital-acquired infections, which can result in prolonged hospital stay, additional surgical procedures and even death [1]. They typically occur within a 30-day post-operative period, and clinical symptoms vary from erythema, swelling and surgical site tenderness to pus discharge and pyrexia [2].

The incidence of SSI varies across surgical specialities and procedures, with clean procedures reporting lower rates and contaminated or dirty operations showing substantially higher risk. These variations reflect differences in inherent bacterial load, surgical approach and underlying pathology [1,2]. In trauma and orthopaedic surgery, mainly arthroplasty procedures, SSIs can lead to repeated surgical procedures and result in significant patient morbidity.

Several aspects can help in infection risk reduction, such as managing patient comorbidities, temperature control in the operating theatre, sterilisation techniques and prophylactic antibiotic use [3]. Many guidelines suggest that SSIs can be prevented partially by using antiseptic agents in combination with alcohol, to give the best and longest effect [4].

Surgical hand antiseptics are a critical component of SSI prevention and act synergistically with patient skin preparation. Surgical scrub solutions, typically based on chlorhexidine, povidone-iodine or alcohol-containing formulations, are used to reduce the resident and transient flora on the hands and forearms of the surgical team before donning and gloving, as well as the surgical site on the patient before incision. This is an essential element of multimodal SSI reduction bundles, alongside antibiotic prophylaxis, where needed, and preoperative skin preparation prior to incision [5]. Systematic evaluations of preoperative infection prevention practices have highlighted that a suboptimal scrub technique or inadequate use of antiseptic scrub solutions can contribute to increased microbial contamination and higher SSI risk, reinforcing the need for adherence to evidence-based surgical scrub protocols [5,6].

SSIs can be costly, both in financial and social terms, and result in an increased hospital stay by about 6.5 days on average, doubling the hospital's financial burden. The estimated annual cost in England alone accounts for about GBP 1 billion [7]. Patients encountering SSIs have a 2-11 times higher risk of mortality in comparison to other patients, and about 77% of these deaths can be directly attributed to SSI and not other factors [8].

The majority of published studies focus on the types of antiseptic agents used in a variety of countries, including the United Kingdom (UK), the United States of America (USA), Italy, Ireland, Spain and Germany, but don’t focus on the application methods or required duration of skin contact or drying before a procedure [9]. There is still no universal disinfectant that has a long-term antiseptic effect or storage, and still no agent with a broad antimicrobial effect [10].

The primary objective of this narrative review is to summarise current evidence on which antiseptic agents and application techniques are most effective in reducing SSIs across common surgical settings. The secondary objectives are to: (1) describe the mechanism of action, advantages and limitations of commonly used agents (povidone-iodine, chlorhexidine, alcohol-based combinations and Sterilium), (2) review evidence on application techniques, preoperative bathing and pre-surgical hand antisepsis, (3) summarise reported adverse effects and practical considerations, including cost where available, and (4) identify key gaps in literature and areas requiring further research.

## Review

Methodology****

This is a narrative review of antiseptic solutions used in surgical skin and hand preparation and highlights their role in preventing SSIs. A literature search was conducted using PubMed, EMBASE and Google Scholar between June 1, 2025, and November 3, 2025, with no initial restriction on publication date.

The search strategy combined terms related to antiseptic agents and surgical infection, including: “antiseptic”, “surgical infection”, “surgical site infection”, “SSI”, “Chlorhexidine”, “chlorhexidine-gluconate”, "povidone”, “iodine”, “povidone-iodine”, “sterillium”, “betadine”, "preoperative skin preparation" and “antiseptic cost”. Reference lists of key articles and recent reviews were also screened to identify additional studies.

Inclusion and Exclusion Criteria

Human studies of any design (randomised controlled trials, cohort studies, case-control studies and narrative or systematic reviews) were eligible if they reported on the use of antiseptic agents or application techniques used for preoperative skin preparation, pre-surgical hand antisepsis or preoperative bathing in relation to SSI outcomes, skin colonisation or microbiological culture outcomes. Studies of animal models, in vitro experiments and articles not available in English were excluded.

Title and abstracts were screened for relevance, and potentially eligible articles were reviewed in full text. Given the narrative design of this review and heterogeneity of study designs, formal risk-of-bias scoring and GRADE (Grading of Recommendations Assessment, Development, and Evaluation) evidence grading were not performed. Instead, key methodological strengths and limitations of influential trials and reviews are described narratively in the relevant sections and results are synthesised qualitatively rather than via meta-analysis. A narrative review approach was chosen because the available evidence spans multiple surgical specialities, uses diverse antiseptic formulations and application techniques and reports SSI outcomes using different definitions and measurement methods. These variations make formal pooling or meta-analysis inappropriate, and a qualitative synthesis is better suited to integrate mechanistic insights and contextual factors across this heterogeneous evidence base.

Normal Healing

Skin, the largest organ in the body, forms a primary barrier to infection, and disruption of this barrier during surgery exposes deeper structures to possible microbial contamination. Normal wound healing involves a sequence of events of inflammatory, proliferative and remodelling phases, and any disturbance in this sequence, such as high bacterial load, can result in SSIs [11,12].

SSI: Burden, Classification and Epidemiology

Surgical procedures are usually divided into four main classifications in terms of their cleanliness, and this helps determine the expected rates of infection. The classifications are clean, clean/contaminated, contaminated and dirty. Clean procedures are usually wounds that have not been previously infected, with no signs of inflammation and are usually primarily closed. The majority of elective procedures fall under this. Clean procedures have a predicted infection risk lower than 2% and the gastrointestinal, respiratory and genitourinary tracts are not entered. Clean/contaminated procedures include those where the gastrointestinal, respiratory and genitourinary tracts are entered, but there is no unexpected contamination apart from normal flora. Contaminated wounds include open fractures; their infection risk is less than 10% but sterility is affected due to this. These operations include spillage from the gastrointestinal tract. Dirty wounds have the highest infection rates and include perforated viscera and patients with wounds having existing infections and necrosis [13].

SSI can be defined as an infection occurring within a 30-day period of a surgical procedure or within one year of implant placement, which remains in situ. It can develop either close to the incision site or be in deep tissues at the surgical site. It is typically caused by bacteria entering through incisions [14]. They can be classified into three main groups: superficial-incisional (which involves the skin and subcutaneous layer), deep-incisional infection (involves muscles or the connective tissue layer of the incision) and deep infection (affecting organs or deep spaces in relation to the incision site) [4].

The Health Protection Agency (HPA), which is an organisation in the UK, is responsible for collecting national SSI data. The only mandatory data collection is post-trauma and orthopaedic surgery. They collected data from a total of 438,678 surgical procedures done in 237 National Health Service (NHS) hospitals between 2006 and 2011. For clean surgeries, they reported SSI rates of 0.6% for knee replacement, 0.8% for hip replacement, 1% for cardiac surgery and 5% post limb amputations. The rates were higher for contaminated surgeries, reaching as high as 10% for large bowel surgery [13].

Jenks et al. published a study in the Journal of Hospital Infection in 2013, which looked at the economic burden of SSIs and the financial consequences of preventative measures. They noted that the average length of hospital stay was 10 days, totalling up to 4694 bed-days over 2 years. The average added cost of SSI was £5,239, and within the 2-year period, this totalled up to £2,491,424. The hospital used in the study was an English National Health Service (NHS) hospital. Between 2000 and 2004, the University College London Hospital NHS had an average cost increase of £4,018 due to SSIs [15].

In 2023, Calderwood et al. published strategies to help prevent SSIs in acute care settings and highlighted practical recommendations to aid with this. In addition to prophylactic antibiotic use, they reclassified decolonisation of cardiothoracic and trauma and orthopaedic surgical patients with anti-staphylococcal solutions, as well as using alcohol-based combinations for preparation of skin prior to commencement of surgery. They highlighted the bactericidal effects of alcohol and identified that the combination of chlorhexidine or iodine with alcohol gave faster and longer-lasting antiseptic effects [8].

Joseph Lister (1827-1912) was the first surgeon to use a skin antiseptic agent. Limb amputations in the early nineteenth century had a mortality rate as high as 50%. Lister started managing wounds with phenol as an attempt to reduce tissue necrosis, which led to infections. Due to his method, there was a dramatic drop in post-operative sepsis at the time, and this was the catalyst for the development of modernised antiseptic agents and techniques, including the sterilisation of instruments, the skin and personal protective equipment such as rubber gloves and surgical scrubs [16].

Antiseptic Agents

Ideal antiseptic agents are able to inhibit microorganism growth and development [17]. They have anti-fungal, anti-viral, anti-protozoal, anti-bacterial and anti-spore properties in addition to being non-toxic, hypoallergenic and non-absorbable, safe after repeated use and have post-application residual activity [18].

Appropriate antiseptic application is considered one of the critical measures used to prevent infections in a surgical setting. The goal is the reduction of microorganisms present on the skin and the reduction of the likelihood of infection [7]. A variety of antiseptics are used, including chlorhexidine, alcohol-based solutions and povidone-iodine. In some instances, a combination of these solutions is used. There is still no unified national protocol for their use across the various surgical sites [1].

Povidone-iodine (Betadine®), chlorhexidine and isopropyl alcohol are amongst the three most common pre-operative antiseptic agents used and are known to have broad-spectrum activity [19].

Povidone-iodine (Betadine®):** **Betadine®, the trade name of povidone-iodine, is a solution made up of slow iodine-releasing agents and has a rapid antimicrobial action that attacks methionine and cysteine, which are amino acids present in microorganisms. It attacks fatty acids and nucleotides, acts by iodination to cause cell membrane lipid oxidation and forms salts with microbial proteins. This solution is particularly effective against *Staphylococcus aureus* [17]. The ‘free’ iodine is the bactericidal agent, and its levels depend on the concentration of Betadine® solution [20].

On a cellular level, Betadine® promotes wound healing, neovascularisation and re-epithelization [17]. Unfortunately, one of the disadvantages identified with its use is having the potential for contamination by *Burkholderia cepacia*, especially if the holding containers are improperly cleaned. It can also be associated with iatrogenic hyperthyroidism or hypothyroidism in all age groups due to the transcutaneous absorption of iodine by the skin, with skin absorption varying in degree from one individual to another [19]. Betadine® is contraindicated in patients diagnosed with thyroid disease and hyperthyroidism, especially patients who have been managed with radioiodine [21].

Widmer et al. conducted a multi-centre, cluster-randomised, investigator-masked, non-inferiority trial comparing povidone-iodine in alcohol with chlorhexidine gluconate in alcohol or preoperative skin preparation in abdominal and cardiac surgery [22]. Among 4,403 patients, the observed SSI rate was 6.8% in the povidone-iodine group and 9.9% in the chlorhexidine group, indicating a modest absolute reduction in SSI with the iodine-based formulation in that setting. Although the study was not primarily designed to demonstrate superiority and was limited to cardiac and abdominal surgeries, it provides important evidence that alcohol-based povidone-iodine can be at least as effective as chlorhexidine-alcohol for SSI prevention in these surgical populations.

Chlorhexidine: Chlorhexidine is an organic cationic biguanide that was synthesised in 1950. It has a molecular configuration and is positively charged, giving it a high skin affinity and making it effective. It acts by binding both to the cell wall and membrane, and acts by inhibiting ATPase and increasing potassium uptake [17]. It binds to the bacterial cell wall, which is negatively charged and attacks the membrane of the bacterial cytoplasm, making it bactericidal [21]. Chlorhexidine acts against Gram-negative and Gram-positive bacteria, viruses and yeasts and can have both a bacteriostatic and bactericidal effect [7]. 

The uses of chlorhexidine are diverse, and this includes both the perioperative settings with alcoholic and aqueous solution preparations to lubricant gels used in urethral catheter insertion, vaginal and rectal examination. It can also be found in mouthwashes, toothpaste, cosmetics and even in contact lens solutions [23].

The wide use of chlorhexidine has resulted in a wider patient base with skin hypersensitivity and allergies [23]. Some of the disadvantages of using chlorhexidine include skin irritation and possible damaging effects on contact with soft tissues, as well as a slow onset of antimicrobial effect [21]. It can result in contact dermatitis through delayed allergic reactions known as type IV reactions, as well as eczema [24].

Chlorhexidine is the only antiseptic that can induce resistance or reduced susceptibility. This can be caused by qac-genes, which are carried by plasmids, allowing for easy transfer between different bacterial species. Decreased susceptibility can lead to a two-fold to four-fold rise in the minimum concentration needed for inhibitory function, which reduces sensitivity to this agent [25]. Studies showed that in the absence of alcohol, chlorhexidine alone is superior to Betadine® alone, as it lasts for a longer period and remains active when it comes in contact with blood or serum [8].

Alcohol-based combinations: Alcohol acts by denaturating bacterial cell wall proteins. It acts against a variety of micro-organisms, tubercle bacilli, fungi, viruses, as well as Gram-negative and Gram-positive bacteria. The most important aspect is the concentration of alcohol, not the type of alcohol used, that determines how effective it will be [7]. Alcohol and alcohol-based solution combinations give the fastest reduction in bacterial load out of all the available antiseptic solutions [25]. 

DuraPrep is an alcohol-based povidone-iodine solution and has a faster reduction of positive skin cultures, lower absolute numbers of cultured organisms and a longer duration of action period. It can therefore be concluded that alcohol-based iodine solutions have a higher efficacy than aqueous-based or only-iodine solutions [19].

ChloraPrep is an alcohol-based, broad-spectrum antiseptic solution, and a strength of 70% alcohol with 2% chlorhexidine has proven effectiveness against both superficial and deep incisional infections [19]. The literature shows that ChloraPrep is at least as effective as povidone-iodine and reduces overall cost in perioperative antisepsis in primary total hip replacements [3].

Despite the rapid onset of action, the downside of using alcohol is a shortened duration of action, which is why it is often used in combination with other solutions. In addition to this, it is known to be highly flammable and adequate drying should be achieved prior to cauterisation use [21]. Some studies have reported the incidence of operating theatre fires due to the use of alcohol-based skin agents, which caused significant harm to both patients and surgical staff. This can be prevented by allowing adequate time for the skin to dry completely [16].

Alcohol is an essential element in skin antisepsis due to its rapid action against microbes. A systematic review of five randomised controlled trials found that chlorhexidine-alcohol formulations had a superior effect on SSI prevention than povidone-iodine solutions. However, other studies did not show this superiority and showed no difference between the actions of the two agents. The main conclusion of all these studies was the importance of practising caution and ensuring the alcohol solution is fully dry, as many operating room fires have been reported [26].

Sterilium® and alcohol hand rubs: Sterillium® is composed of n-propanol, iso-propanol and mecetronium ethosulphate as well as auxiliary ingredients such as emollients [27]. It is the first hand gel made up of 85% ethanol [28]. One advantage of its use is that it does not currently have any identified clinically relevant potential for sensitisation or dermal irritation, and it has shown superior dermal tolerance in comparison to other agents [27].

It is the only hand-rub agent in literature so far that meets the required criteria for bactericidal efficacy. In comparative studies, Sterilium achieved greater reduction in bacterial counts than other alcohol-based hand antiseptic solutions, and one trial reported a statistically significant difference in colony count reduction (p<0.001) compared with Hibiscrub [29,30]. 

Application Technique (Skin Preparation)

Skin is the first barrier protecting against infection in the human body; it is therefore essential to ensure this barrier is protected prior to invasive procedures. Appropriate preparation is linked to the type of solution being used, the technique used (such as circular application versus back-and-forth painting application), as well as the time given for the solution to dry prior to commencing needle insertion or skin incision [31].

Although the type of antiseptic used is important, the correct application method of that antiseptic is of equal importance; unfortunately, this has not been adequately researched. In traditional methods, the majority of antiseptic applications have been done using concentric circles starting at the intended incision site and going outwards. This is particularly important for aqueous-based antiseptic solutions, which need drying time to avoid the reintroduction of microorganisms and contaminants to areas which have already been cleansed [9]. The majority of existing guidelines do not focus on or mention the importance of the application method of these antiseptic agents. An exception would be Spanish guidelines, which recommend the use of an applicator with a back-and-forth application method lasting 30 seconds [9].

In the USA, a study was published by Lundberg et al. in 2016, where they recruited 30 subjects who do routine skin preparation for surgery and were asked to fill a questionnaire assessing familiarity with the most common antiseptic agents used: povidone-iodine and chlorhexidine gluconate during scrubbing and painting of patients. Despite having adequate experience in the operating room, none of the subjects were able to answer all of the questions correctly. The study showed a lack of compliance with protocol when performing preoperative antisepsis, and none of them completed all of the recommended steps for povidone-iodine or chlorhexidine application [7].

Circular application begins at the centre of the circle and moves outwards. It is recommended to use a separate application and repeat it for an additional two times. Although it does not guarantee that the skin is aseptic after the third application, it still indicates that a disinfecting solution was used, and the skin at that point is said to be clean [31].

The back-and-forth or paint-brush application allows some friction at the centre of the surgical side, and if this method is used for a specified length of time, it is able to clean through the top five dermal layers of skin and remove a large portion of bacterial load found in the dermal layer of skin [31].

Summary of Common Agents

Skin is not a sterile organ and contains thousands of microorganisms residing in it naturally, which contribute to maintenance colonies that inhibit the growth of fungal and yeast infections [32].

Dockery et al. published a review article in 2021, which summarised the most recent publications on preparation solutions and SSIs [19]. The lower limb has a higher rate of infection than upper limb, with foot and ankle surgery infection ranging from 2.8% to 13.2%, commonly isolating coagulase-negative staphylococci, *Staphylococcus aureus*, methicillin-resistant *Staphylococcus aureus* (MRSA), these are the same microorganisms affecting hip and knee arthroplasty and their revisions, with a rate of 2% affecting the initial arthroplasty procedures and 4.5% affecting revision surgeries. Shoulder surgeries have an infection rate of 1-10% [19], and the commonly isolated organisms in culture are *Staphylococcus* and* Propionibacterium acnes* [33]. With all these procedures, the most commonly used and recommended antiseptic agents are ChloraPrep, DuraPrep or Betadine® [19].

A study was published by Saltzman et al. in 2009, in The Journal of Bone & Joint Surgery, looking into the types of native bacteria present naturally in the shoulder region and assessing the efficacy of three antiseptic solutions commonly used in pre-operative skin preparation in eradicating bacteria from the shoulder. Overall, 150 patients were evaluated, with one of the three solutions, ChloraPrep, Betadine® or DuraPrep, randomly selected for skin preparation and skin swabs taken for both aerobic and anaerobic cultures. The most common microorganisms isolated were coagulase-negative *Staphylococcus* and *Propionibacterium acnes*. The study noted that Betadine® was less effective in the elimination of coagulase-negative Staphylococcus but found no notable difference between the three solutions in the elimination of *Propionibacterium acnes* [34].

In France, another study was published by Rougereau et al. in 2022, where prospective data collection was done for all total hip arthroplasties (THAs) at a university hospital between 1 November and 31 December 2020, and surgeons were randomly assigned an antiseptic, either ChloraPrep or Betadine®, before the study began. They looked into a variety of factors, including the cost of the product, disposal of waste and occupancy time of each protocol in the operating room for each patient. They found that ChloraPrep took marginally less time to apply and cost less than Betadine® (€46.8 was the cost of ChloraPrep and €155 was the cost of Betadine®) [3].

Allergic reactions and hypersensitivity to povidone-iodine and chlorhexidine are rare but have been mentioned in the literature. There are fewer than a hundred cases published in the literature of severe anaphylaxis cases. For chlorhexidine, patients can encounter contact dermatitis, hypersensitivity, and in rare, severe cases, anaphylactic shock can occur. For povidone-iodine, it can result in allergic contact dermatitis, but this is rare. It can also result in immediate immunologic reactions such as urticaria or anaphylaxis [25].

Pre-operative Bathing With Antiseptics

The practice of showering or bathing with antiseptics is widely spread and aims to prevent SSIs. The goal is to ensure the skin is as clean as possible before surgery by washing off transient and resident flora. Some commonly used agents include 4% chlorhexidine, also known as Hibiscrub [32]. 

Two per cent ChloraPrep (which is made up of 2% chlorhexidine gluconate and 70% isopropyl alcohol) has been proven more effective than DuraPrep (which is made up of 0.7% iodophor and 74% isopropyl alcohol). Shoulder surgeries are mostly caused by native skin flora, which is why a home skin preparation protocol aids in the reduction of these infections, reducing the load of cutaneous bacterial pathogens and helping prevent SSIs [33].

In a systematic review published by Webster & Osborne in 2015, they identified a 10-year prospective study that performed surveillance on the rate of SSIs amongst patients made to shower preoperatively with hexachlorophene and compared to patients who did not. This was a study published by Cruise et al. in 1980, which found a lower rate of SSIs in those who showered with hexachlorophene preoperatively. They also identified an additional study published by Colling et al. in 2014, in which a retrospective study was carried out to measure the incidence of SSI post total knee arthroplasty or THA over a two-year period. They noted no difference in SSI incidence overall but noted a reduction in the incidence of *Staphylococcus aureus* and MRSA [32].

A meta-analysis and systematic review of clinical trials, both randomised and non-randomised, was carried out by Lefebvre et al. in 2014 and published in The Journal of Hospital Infection and looked at pre-surgical scrubbing before painting, analysing if it carried any additional value in reducing SSI incidence. They reviewed 7 main trials, with a total of 1,652 patients for positive skin culture outcomes. They did not note any significant difference in positive cultures in patients who were scrubbed before painting versus those who were only painted. They recommended additional research to prove this. They believe that if the surgical site skin looks clean or the patient had a pre-operative shower, there is no need for additional pre-scrubbing [35].

*Pre-surgical Hand Antisepsis*****

In a surgical setting, the team members performing the pre-surgical hand antisepsis (or scrubbing techniques) are the scrub nurse and the operating surgeons. Scrub nurse and surgeon hands have been identified as an infection source. Common nosocomial infections are due to MRSA, *Actinobacter baumannii*, *Staphylococcus aureus* and *Pseudomonas aeruginosa*. Traditionally, the antisepsis technique for hands included an aqueous scrub solution using Betadine® or chlorhexidine, which can be used with or without a surgical sponge. In more recent years, some healthcare institutions began using alcohol-based hand rubs as an alternative to this [36].

In 2009, the World Health Organisation (WHO) published guidelines on healthcare-setting hand hygiene and recommended at least a 2-5-minute duration for a surgical antisepsis scrub for the hands and forearms [37]. This guideline, however, does not highlight the technique of scrubbing or the agents used.

In Egypt, a prospective study was done with the goal of comparing the antiseptic effects of Sterillium® and Betadine® on the microbial load on the hands of surgeons and scrub nurses. The sample size was 54 surgeons and 44 scrub nurses. The mean microbial load post Betadine® scrubbing was 4.06, and post Sterilium® hand rub was 0.15, showing that Sterilium® hand rub, at the time, was proven to be more effective in reducing hand microbial load before surgery [36]. 

There are two main techniques for surgical hand scrubbing, known as the 4-minute scrub (4MS) and the 10-stroke scrub (10SS). Both have proven to be effective in appropriate pre-surgical hand antiseptic technique [37]. Table 1 shows a summary of the steps involved in the 4MS and 10SS techniques.

**Table 1:** The 4MS and 10SS surgical scrubbing techniques Source: Aslan et al., 2023 [37] Key: 4MS = 4-min scrub, 10SS= 10-stroke scrub

Technique	Steps involved
4MS	The hand and forearm must be scrubbed evenly without any specific method. Each arm gets scrubbed for approximately two minutes.
10SS	The technique has a checklist that must be adhered to; it divides the upper limb into regions (palm, dorsum of hand, sides of fingers, inner-outer wrist and forearm, followed by elbow). A back-and-forth method of sponge application is used for each part and repeated 10 times. Once a region has been scrubbed ten times, it doesn’t get repeat scrubs.

In terms of hand washing duration, a literature review evaluated four randomised controlled trials, which compared the durations of surgical team antisepsis. One study did not show any difference between scrubbing for two or three minutes when compared to hand washing with soap and water for a full minute. Another study showed that washing hands for a full minute followed by using alcohol hand rub for three minutes was superior to only using an alcohol hand rub for five minutes. A third study showed that spending equal time using chlorhexidine or betadine showed similar results [26]. The literature varies, and there is still no consensus on the appropriate duration that can be used as a universal guideline.

Summary/synthesis

Across available studies, alcohol-based preparations consistently outperform aqueous formulations. Chlorhexidine-alcohol combinations are commonly used in trauma and orthopaedics and in clean procedures, while alcohol-based povidone-iodine shows comparable or superior outcomes in abdominal or cardiac surgeries. The available evidence does not support a single antiseptic solution as universally superior for all procedures and settings. Several trials and reviews suggest that chlorhexidine-alcohol formulations reduce SSI rates more effectively than aqueous povidone-iodine in clean and clean-contaminated surgery [13,18,26]. In contrast, the Widmer et al. trial in cardiac and abdominal surgery found lower SSI rates with alcohol-based povidone-iodine than with chlorhexidine-alcohol [22]. These findings are not necessarily contradictory when differences in formulation, surgical site and baseline infection risk are considered. Similarly, studies of preoperative showering and scrubbing before painting dress different steps in the antiseptic pathway; some show benefit in bacterial load reduction or organism-specific outcomes, while others find no additional advantage beyond standard painting [32,35]. Overall, the strongest and most consistent preference is to alcohol-based combinations over purely aqueous solutions, with the choice between chlorhexidine- and iodine-based formulations depending on surgical context, patient factors and hospital protocols. 

Table 2 provides a summary of key studies evaluating commonly used antiseptic agents, their comparative effectiveness and their implications for SSI prevention.

**Table 2:** Summary of key studies evaluating antiseptic agents and surgical site infection outcomes

Study	Year of publication	Design/population	Agents / Focus	Key findings	Implications
Widmer et al. [22]	2024	Multicentre cluster-randomised trial for cardiac and abdominal surgery	Povidone-iodine in alcohol versus chlorhexidine-alcohol	SSI 6.8% versus 9.9%, favouring povidone-iodine in alcohol	Alcohol-based povidone-iodine is at least as effective as, and potentially superior to, chlorhexidine-alcohol in some high-risk settings
Dockery et al. [19]	2021	Narrative review (orthopaedic surgery)	Preoperative skin preparation agents	Alcohol-based combinations consistently outperform aqueous formulations; no single superior agent	Supports preference for alcohol-based agents tailored to the surgical site
Saltzman et al. [34]	2009	Randomised study on shoulder surgery	ChloraPrep versus Betadine versus DuraPrep	Betadine is less effective against coagulase-negative staphylococci, with similar efficacy to *Propionibacterium acnes*	Highlights site-specific differences in antiseptic performance
Rougereau et al. [3]	2022	Prospective cost-effectiveness analysis for total hip arthroplasty	ChloraPrep versus Betadine	ChloraPrep is cheaper (€46.8 versus €155) and marginally faster to apply	Cost and workflow considerations favour chlorhexidine-alcohol
Dumville et al. [13]	2015	Systematic review on clean surgery	Preoperative skin antiseptics	Several trials show lower SSI rates with chlorhexidine-alcohol than aqueous povidone-iodine	Supports chlorhexidine-alcohol in many cleaning procedures
Webster & Osborne [32]	2015	Systematic review	Preoperative antiseptic bathing	Mixed evidence for SSI reduction, some improvement in bacterial load	Useful mainly in selected high-risk scenarios
Lefebvre et al. [35]	2014	Meta-analysis	Scrubbing before painting versus painting alone	No significant reduction in positive cultures with additional scrubbing	Painting alone is adequate when the skin is clean

Limitations

While interpreting these findings, some important considerations should be noted. The published evidence varies considerably in study design, antiseptic formulations, application techniques and SSI outcome definitions, limiting direct comparability between studies. These differences highlight the need for greater harmonisation of research methodology in this field. Additionally, non-English publications and grey literature were not included, which may have resulted in the omission of potentially relevant data.

Recommendations 

While research on the types of antiseptic agents used and their mechanism of action is abundant, there is still a lack of published data about the appropriate technique, the number of layers or coatings and variations in application-time or drying-time for antiseptic agents and whether they cause higher or lower SSIs.

Alcohol-based skin preparations are superior and preferred over aqueous solutions alone for most clean and clean-contaminated surgeries. In trauma and orthopaedic surgeries, mainly arthroplasty operations, the use of chlorhexidine-alcohol is widely encouraged.

In patients without known iodine-related thyroid disease or history of povidone-iodine reactions, chlorhexidine-based solutions are generally preferred, whereas in patients with documented chlorhexidine hypersensitivity, povidone-iodine-based solutions are safer [21,25].

Although preoperative bathing can help reduce bacterial load, the evidence of SSI reduction remains inconsistent, and it is recommended to consider its use in high-risk procedures. Current evidence does not support universal mandatory protocols; it may be most useful in high-risk populations or procedures [32,33].

Whichever agents are used, adherence to manufacturer instructions, adequate coverage of the surgical field, and sufficient drying time remain critical steps in the reduction of SSIs.

The author of this paper recommends a more nation-wide or international multi-centric cohort study aiming to determine the effect of the above, to compare data and help reduce the SSI burden.

## Conclusions

SSIs remain a major cause of morbidity and cost across all surgical specialities. Alcohol-based antiseptic solutions, either chlorhexidine-alcohol or povidone-iodine-alcohol combinations, show the best evidence for reducing SSI risk; however, there is no single solution seen as superior for all procedures. Technique, coverage and adequate drying times are all essential to ensure the appropriate use of these agents. Although extensive research exists on antiseptic agents and their mechanism of action, research is still lacking on clear guidance on the optimal application technique, timing and standardised protocol. Further, multi-centre studies are required to address these gaps and support a more unified practice to ensure appropriate SSI incidence reduction.

## References

[1] Du Q, Wu S, Jin Z, Ni L (2025). Summary of the best evidence for the use of antiseptics at various surgical sites to prevent postoperative infections. Front Med (Lausanne).

[2] Liu G, Ma L (2024). Factors influencing surgical site infections and health economic evaluation in patients undergoing robot-assisted radical resection for colorectal cancer. J Cancer Res Ther.

[3] Rougereau G, Chatelain L, Terracher R, Zadegan F, Ollat D (2022). Surgical solutions for preoperative skin preparation in total hip arthroplasty: a cost-effectiveness analysis of Betadine® and Chloraprep™. Orthop Traumatol Surg Res.

[4] Seidelman JL, Mantyh CR, Anderson DJ (2023). Surgical site infection prevention. A review. JAMA.

[5] Mohammed Al-Aamri HH, Nair AS, Al Sawafi KM, Al Sharji I, Al Jabri A (2024). Influence of surgical scrubs outside the operation theatre on post-operative infections - a systematic review. Indian J Anaesth.

[6] Rezaei AR, Zienkiewicz D, Rezaei AR (2025). Surgical site infections: a comprehensive review. J Trauma Inj.

[7] Lundberg PW, Smith AA, Heaney JB, Wimley WC, Hauch AT, Nichols RL, Korndorffer JR Jr (2016). Pre-operative antisepsis protocol compliance and the effect on bacterial load reduction. Surg Infect (Larchmt).

[8] Calderwood MS, Anderson DJ, Bratzler DW (2023). Strategies to prevent surgical site infections in acute-care hospitals: 2022 update. Infect Control Hosp Epidemiol.

[9] Casey AL, Badia JM, Higgins A, Korndorffer J, Mantyh C, Mimoz O, Moro M (2017). Skin antisepsis: it's not only what you use, it's the way that you use it. J Hosp Infect.

[10] Dymnikova NS, Kusnetsov OY, Moryganov AP (2023). Development of the formulation of antiseptics and disinfectants based on silver nanoparticles. Russ J Gen Chem.

[11] Mohamed SA, Hargest R (2021). Surgical anatomy of the skin. Surgery (Oxford).

[12] Gushiken LF, Beserra FP, Bastos JK, Jackson CJ, Pellizzon CH (2021). Cutaneous wound healing: an update from physiopathology to current therapies. Life (Basel).

[13] Dumville JC, McFarlane E, Edwards P, Lipp A, Holmes A, Liu Z (2015). Preoperative skin antiseptics for preventing surgical wound infections after clean surgery. Cochrane Database Syst Rev.

[14] Habtie TE, Feleke SF, Terefe AB, Alamaw AW, Abate MD (2025). Nurses' knowledge and its determinants in surgical site infection prevention: a comprehensive systematic review and meta-analysis. PLoS One.

[15] Jenks PJ, Laurent M, McQuarry S, Watkins R (2014). Clinical and economic burden of surgical site infection (SSI) and predicted financial consequences of elimination of SSI from an English hospital. J Hosp Infect.

[16] Hemani ML, Lepor H (2009). Skin preparation for the prevention of surgical site infection: which agent is best?. Rev Urol.

[17] Iacopi E, Giangreco F, Piaggesi A (2023). Principles of antiseptic treatments. Pearls and Pitfalls in Skin Ulcer Management.

[18] Sidhwa F, Itani KM (2015). Skin preparation before surgery: options and evidence. Surg Infect (Larchmt).

[19] Dockery DM, Allu S, Vishwanath N (2021). Review of pre-operative skin preparation options based on surgical site in orthopedic surgery. Surg Infect (Larchmt).

[20] Durani P, Leaper D (2008). Povidone-iodine: use in hand disinfection, skin preparation and antiseptic irrigation. Int Wound J.

[21] Letzelter J, Hill JB, Hacquebord J (2019). An overview of skin antiseptics used in orthopaedic surgery procedures. J Am Acad Orthop Surg.

[22] Widmer AF, Atkinson A, Kuster SP (2024). Povidone iodine vs chlorhexidine gluconate in alcohol for preoperative skin antisepsis. A randomized clinical trial. JAMA.

[23] Rose MA, Garcez T, Savic S, Garvey LH (2019). Chlorhexidine allergy in the perioperative setting: a narrative review. Br J Anaesth.

[24] Beaudouin E, Kanny G, Morisset M (2004). Immediate hypersensitivity to chlorhexidine: literature review. Eur Ann Allergy Clin Immunol.

[25] Jolivet S, Lucet JC (2019). Surgical field and skin preparation. Orthop Traumatol Surg Res.

[26] Tokarski AT, Blaha D, Mont MA (2014). Perioperative skin preparation. J Orthop Res.

[27] Kampf G, Muscatiello M (2003). Dermal tolerance of Sterillium, a propanol-based hand rub. J Hosp Infect.

[28] Kampf G, Rudolf M, Labadie JC, Barrett SP (2002). Spectrum of antimicrobial activity and user acceptability of the hand disinfectant agent Sterillium Gel. J Hosp Infect.

[29] Kampf G, Kapella M (2003). Suitability of Sterillium GelTM for surgical hand disinfection. J Hosp Infect.

[30] Pietsch H (2001). Hand antiseptics: rubs versus scrubs, alcoholic solutions versus alcoholic gels. J Hosp Infect.

[31] Stonecypher K (2009). Going around in circles: is this the best practice for preparing the skin?. Crit Care Nurs Q.

[32] Webster J, Osborne S (2015). Preoperative bathing or showering with skin antiseptics to prevent surgical site infection. Cochrane Database Syst Rev.

[33] Murray MR, Saltzman MD, Gryzlo SM, Terry MA, Woodward CC, Nuber GW (2011). Efficacy of preoperative home use of 2% chlorhexidine gluconate cloth before shoulder surgery. J Shoulder Elbow Surg.

[34] Saltzman MD, Nuber GW, Gryzlo SM, Marecek GS, Koh JL (2009). Efficacy of surgical preparation solutions in shoulder surgery. J Bone Joint Surg Am.

[35] Lefebvre A, Saliou P, Mimoz O (2015). Is surgical site scrubbing before painting of value? Review and meta-analysis of clinical studies. J Hosp Infect.

[36] Ali R, Eldegla H, Abdelraoof S (2022). Comparison of the antiseptic effects of Betadine and Sterillium on microbial load of surgical hands. Mansoura Nurs J.

[37] Aslan L, Subasi O, Mizikoglu D (2023). A new checklist surgical hand scrub to replace time-based methods - a pixel intensity analysis. Surgeon.

